# Biocatalyzed Synthesis of Flavor Esters and Polyesters: A Design of Experiments (DoE) Approach

**DOI:** 10.3390/ijms22168493

**Published:** 2021-08-06

**Authors:** Filippo Fabbri, Federico A. Bertolini, Georg M. Guebitz, Alessandro Pellis

**Affiliations:** 1 Department of Agrobiotechnology, Institute of Environmental Biotechnology, University of Natural Resources and Life Sciences, Vienna, Konrad Lorenz Strasse 20, 3430 Tulln an der Donau, Austria; filippo.fabbri@boku.ac.at (F.F.); federico.bertolini@boku.ac.at (F.A.B.); guebitz@boku.ac.at (G.M.G.); 2 Austrian Centre of Industrial Biotechnology, Konrad Lorenz Strasse 20, 3430 Tulln an der Donau, Austria

**Keywords:** bioplastics, polyesters synthesis, flavor esters synthesis, hydrolytic enzymes, design of experiments, closure of the carbon cycle

## Abstract

In the present work, different hydrolases were adsorbed onto polypropylene beads to investigate their activity both in short-esters and polyesters synthesis. The software MODDE^®^ Pro 13 (Sartorius) was used to develop a full-factorial design of experiments (DoE) to analyse the thermostability and selectivity of the immobilized enzyme towards alcohols and acids with different chain lengths in short-esters synthesis reactions. The temperature optima of *Candida antarctica* lipase B (CaLB), *Humicola insolens* cutinase (HiC), and *Thermobifida cellulosilytica* cutinase 1 (Thc_Cut1) were 85 °C, 70 °C, and 50 °C. CaLB and HiC preferred long-chain alcohols and acids as substrate in contrast to Thc_Cut1, which was more active on short-chain monomers. Polymerization of different esters as building blocks was carried out to confirm the applicability of the obtained model on larger macromolecules. The selectivity of both CaLB and HiC was investigated and best results were obtained for dimethyl sebacate (DMSe), leading to polyesters with a M_w_ of 18 kDa and 6 kDa. For the polymerization of dimethyl adipate (DMA) with BDO and ODO, higher molecular masses were obtained when using CaLB onto polypropylene beads (CaLB_PP) as compared with CaLB immobilized on macroporous acrylic resin beads (i.e., Novozym 435). Namely, for BDO the M_n_ were 7500 and 4300 Da and for ODO 8100 and 5000 Da for CaLB_PP and for the commercial enzymes, respectively. Thc_Cut1 led to polymers with lower molecular masses, with M_n_ < 1 kDa. This enzyme showed a temperature optimum of 50 °C with 63% of DMA and BDO when compared to 54% and 27%, at 70 °C and at 85 °C, respectively.

## 1. Introduction

Plastic has succeeded to enter in each and every aspect of our lives, providing a cheap, lightweight, and inexpensive material having outstanding properties. However, its versatility represents a double-edge sword; the extraction and processing of the raw materials (mainly petrochemical origin) to obtain plastics have, in fact, a huge environmental impact [1]. Moreover, there is also an increasing amount of non-degradable plastic waste that often pollutes the environment and a high production of CO_2_ and toxins that are released into the atmosphere following potential incineration [2,3]. In particular, the analysis of the United Nations Environment Programme (UNEP) indicated that over 75% of the cost of natural capital (considered as the set of natural assets necessary for the existence of productive activities) of the plastic use in goods of consumption (estimated at 75 billion dollars per year) derives from the extraction of raw materials for plastic and its manufacture [4]. Although the annual European production has been steady in the past decade, the global production is constantly increasing and reached 368 million tons in 2019 [5]. All these issues, in combination with the major problems that have emerged in the last 40 years, such as climate change, depletion of oil fields, and environmental pollution, highlight the importance of moving towards more sustainable, bio-based, and environmentally friendly processes across a wide range of different industries.

Enzymes represent a solid alternative for polymer synthesis, offering a concrete response to the challenge of combining benign conditions with high selectivity and efficient activity [6]. In fact, enzyme utilization is a sustainable alternative that permits us to apply milder reaction conditions regarding temperature (T < 100 °C), avoiding toxic catalysts such as tin and titanium, that are well-established, for example, in polycondensation reactions [7,8]. Moreover, recent innovations allow the biotechnological production of bio-based monomers from renewable sources, enabling the replacement of petrochemical building blocks [9,10]. Among polymers, polyesters are a widely used class with applications ranging from food packaging to devices used in medical fields and from car components to children’s toys. The most investigated areas of enzymatic synthesis in the production of polyesters are via both polycondensation (transesterification) and ring opening polymerizations (ROPs) [9,11]. These procedures have been widely investigated and optimized thanks to the discovery and commercial availability of *Candida antarctica* lipase B (CaLB), both in its free and immobilized formulation, known as Novozym 435 (immobilized on macroporous acrylic resin). Furthermore, other enzymes belonging to the α,β-hydrolases superfamily have been largely explored as biocatalysts for polyesters synthesis, showing promising results, as for instance in the case of the well-known *Humicola insolens* cutinase (HiC) [12,13] or for the more recent *Thermobifida cellulosilytica* cutinase 1 (Thc_Cut1) [14].

Immobilization of enzymes represents a key-point requirement for industrial-scale synthesis. In fact, in numerous cases, it has been proven that enzyme immobilization not only enables subsequent recyclability and simple separation of the product in the downstream processes, but also leads to improved selectivity and specificity as well as stability for the biocatalyst [15,16,17]. In particular, regarding hydrolytic enzymes, Pellis et al. (2016) described covalent immobilization of lipases and cutinases on epoxy resins and the use of these formulations for polycondensation reactions, highlighting outstanding stability and recyclability for the obtained formulations even after 10 cycles [6]. In general, among the different immobilization techniques, physical adsorption is a very simple, cheap, and convenient approach, since the protein molecules adhere to the surface of the carrier matrix or the pores in the case of mesoporous materials by a combination of hydrophobic interactions, H-bondings, van der Waal forces, and electrostatic interactions [18]. In fact, Weinberger et al. (2018) reported successful immobilization of CaLB through physical adsorption onto polypropylene beads and the subsequent bio-synthesis of polyesters, obtaining high M_w_ products (4 kDa) [16]. In particular, in addition to polyesters, immobilized hydrolytic enzymes are already used at the industrial-scale to produce environmentally-friendly short-chain esters like modified triacylglycerols, emulsifiers, peptides, and oligosaccharides under mild operating conditions, achieving high product purity due to reduced side reactions. Such products are used in the food industry, fragrances, cosmetics, or the pharmaceutical industry [19,20]. A boost in this field was provided by the pioneering work by Gillies et al. (1987) that first described the biosynthesis of different flavour esters by *Candida cylindracea* lipase immobilized on silica gel, achieving >90% conversion rate for ethyl butyrate, ethyl octanoate, and other flavor esters [21].

Among the many studies that were performed during the past 30 years on the biocatalyzed synthesis of polyesters, despite few exceptions, we found a general lack of information regarding the main biocatalyst chain-length selectivity and thermostability in aliphatic polyesters synthesis. Pellis et al. (2018) reported immobilized CaLB (N435) activity towards C_4_–C_10_ diesters and C_4_–C_8_ diols, obtaining the best combination using dimethyl adipate and 1,8-octanediol at 85 °C, leading to a M_n_ of 7100 Da [7]. Regarding HiC, Feder and Gross (2010) investigated polycondensations of different linear diacids and various diols using this enzyme immobilized on Amberzyme oxirane resin at 70 °C, leading to the conclusion that HiC expresses its preference towards long-chain compounds, observing a M_n_ of 6600 Da using dimethyl sebacate and 1,8-octanediol [22]. On the other hand, Thc_Cut1 showed a substrate preference for C_4_–C_6_ diesters and diols at 50 °C when immobilized using a His-tag method on different carriers (opal, coral, and amber beads), resulting in a polyester with a molecular weight of 900 Da (M_w_) [23]. Higher M_w_ (~1900 Da) was obtained using the biocatalyst covalently immobilized on epoxy resins [6]. In addition, a multivariate factorial design combined with computational investigations performed using Thc_Cut1 immobilized on a carrier based on milled rice husk described this enzyme stability in solvent-free polycondensations at 50 °C, shedding light on its structural and functional elements [14].

In this study, after immobilization of different hydrolytic enzymes onto polypropylene beads, the software MODDE^®^ Pro 13 (Sartorius) was used to develop a full-factorial design of experiments (DoE) in order to analyze immobilized enzyme thermostability and selectivity towards alcohols and acids with different chain lengths in short-esters synthesis reactions. MODDE^®^ optimized the coefficients involved in the DoE (temperature, alcohol length, acid length, and reaction time) to obtain a visual-model that defined the optimal synthetic profile of the biocatalysts. The results for the commercial enzymes were compared to the data available in the literature, while it was the first time that a complete optimization was evaluated for Thc_Cut1. Afterwards, different bio-based building blocks, such as dimethyl adipate (DMA), dimethyl itaconate (DMI), and dimethyl sebacate (DMSe) in combinations with the diols 1,4-butanediol (1,4-BDO) and 1,8-octanediol (1,8-ODO), were used as monomers for biocatalyzed synthesis of polyesters through polycondensation reactions, using the previously characterized immobilized hydrolytic enzymes that were found suitable for polycondensations. In particular, using CaLB, interesting polyesters containing DMA were synthesized, showing high monomer conversion rates (>90%), average molecular weight (M_w_) and average number molecular weight (M_n_), even higher than commercial iCaLB (M_n_ 7500 vs. 4300 Da using BDO and 8100 vs. 5000 Da using ODO).

## 2. Materials and Methods

### 2.1. Chemicals and Reagents

Polypropylene beads (Accurell MP1000 surface area of 55,985 m^2^ g^−1^, particle density of 1.993 cm^−3^, and particle diameter of <1500 mm) were purchased from 3M Deutschland GmbH (Wuppertal, Germany). Dimethyl adipate (DMA, >99%), dimethyl sebacate (DMSe, >99%), 1,4-butanediol (BDO, >99%), 1,8-octanediol (ODO, >98%), butyric acid (>99%), 1-butanol (>99%), 1-octanol (>99%), 1-dodecanol (>99%), lauric acid (>99%), 2-methyl-2-butanol (>99%), 4-nitrophenol (>99%), para-nitrophenyl butyrate (*P*-NPB), Na_2_HPO_4_ (>98%), NaH_2_PO_4_ (>98%), and toluene (>99.8%) were purchased from Sigma-Aldrich. Dimethyl itaconate (DMI, 98%) was purchased from TCI chemicals (Vienna, Austria). Octanoic acid (>98%) and 2-methyltetrahydrofuran (MeTHF, >99%) were purchased from Alfa Aesar (Kandel, Germany). Chloroform (for HPLC, >99.8%) was purchased from VWR Chemicals (Wien, Austria).

All chemicals and solvents were used as received if not otherwise specified.

### 2.2. Enzymes

The recombinant *Thermobifida cellulosilytica* cutinase 1 (Thc_Cut1) was produced and purified as previously described [24]. The organism used for the expression was *E. coli*. Novozym^®^ 435 (product code: L4777) containing *Candida antarctica* lipase B immobilized on macroporous acrylic resin beads (iCaLB), Lipozyme CaLB, *Thermomyces lanuginosus* lipase (TLL), and *Aspergillus niger* lipase (AnL) and were acquired from Sigma-Aldrich (Vienna, Austria). *Humicola insolens* cutinase (HiC, CAS 9001-62-1) was obtained from Strem Chemicals, Inc. (Newburyport, MA, USA).

### 2.3. Enzymes Immobilization on Polypropylene Beads

2.0 g of polypropylene beads were accurately weighed in a 50-mL centrifuge tube and washed with 20 mL of acetone at 600/700 mbar for 5 min to remove the air and enable the enzyme binding also in the bead’s cavities. This procedure was repeated three times. Afterwards, the beads were rinsed with 20 mL of ddH_2_O (once) and with 20 mL of the immobilization buffer, 0.1 M Na_2_HPO_4_/NaH_2_PO_4_ buffer at pH 8 (twice). The washing steps were carried out using a blood rotator set at 30 rpm. After the washings, 45 mL of 0.44 mg mL^−1^ (1% *w w*^−1^ enzyme/beads), 1.11 mg mL^−1^ (2.5% *w w*^−1^ enzyme/beads), or 2.22 mg mL^−1^ (5% *w w*^−1^ enzyme/beads) were added to the beads at 21 °C and left to react for 24 h on a blood rotator set at 30 rpm. Samples were withdrawn over time to monitor the reaction progression by analyzing the residual enzyme activity and residual protein concentration in the supernatant. After 24 h, the beads were filtrated using a paper filter, rinsed 3 times with 10 mL of the immobilization buffer and air-dried for 3 days at 21 °C before further use. The hydrolytic activity of the immobilized enzyme was checked using an adapted version of the esterase activity assay reported in Section 2.5.

### 2.4. Determination of the Extinction Coefficient of Nitrophenol in Na_2_HPO_4_/NaH_2_PO_4_ Buffer

A solution containing 10 mmol of 4-nitrophenol in 10 mL of 0.1 M Na_2_HPO_4_/NaH_2_PO_4_ buffer at pH 8 was prepared. Further dilutions with the same buffer were prepared. The absorbance of 220 μL in different solutions was measured at 405 nm (in triplicates) at 30 °C with a Tecan Reader (Tecan, Grödig, Austria) using a 96-well microtiter plate (Greiner 96 Flat Bottom Transparent Polystyrene). A blank was included using the buffer. The extinction coefficient of nitrophenol is equal to the slope of the obtained calibration curve (Figure S6 in ESI).

### 2.5. Esterase Activity Assay

Esterase activity was determined by measuring the amount of para-nitrophenyl butyrate (*p*-NPB) enzymatically hydrolyzed at 30 °C; 200 μL of the substrate solution (consisting of 86 μL of *p*-NPB and 1000 μL of 2-methyl-2-butanol) was mixed with 20 μL of the enzyme diluted in buffer. The catalytic activity, which corresponds to the increment of the absorbance at 405 nm due to the hydrolytic release of p-nitrophenol (ε 405 nm), was monitored over 5 min in cycles of 18 s with a Tecan Reader (Tecan, Grödig, Austria) using 96-well microtiter plates (Greiner 96 Flat Bottom Transparent Polystyrene). A blank was included using the buffer. The activity was calculated in units (U), where 1 unit is defined as the amount of enzyme required to hydrolyze 1 μmol of substrate per minute.

### 2.6. Protein Concentration Determination

Protein concentration was measured at 30 °C using Bio-Rad solution (Coomassie brilliant blue G-250 dye, Bio-Rad, Vienna, Austria) diluted 1:5 with ddH_2_O. Bovine serum albumin was used as a standard. The supernatant was diluted in 0.1 M Na_2_HPO_4_/NaH_2_PO_4_ buffer at pH 8. Then, 10 μL of the sample were put in the well and 200 μL of 1:5 BioRad solution were added (in triplicate). The solutions were incubated at 21 °C for 5 min at RT 400 rpm. The absorbance at 595 nm due to the dye binding to primarily basic (especially arginine) and aromatic amino acid residues (ε 595) was measured over 5 min in cycles of 18 s at 30 °C with a Tecan Reader (Tecan, Grödig, Austria) using a 96-well microtiter plate (Greiner 96 Flat Bottom Transparent Polystyrene). A blank was included using the immobilization buffer. The protein concentration was calculated using a calibration curve obtained using a BSA standard (Bovine Serum Albumin protein standard, 2 mg/mL, Sigma-Aldrich).

### 2.7. Planning of the Design of Experiments (DoE)

The full factorial design was planned using the software MODDE^®^ Pro 13 (Sartorius). The considered independent variables were temperature (50 °C, 70 °C, and 90 °C), alcohol chain length (C_4_, C_8_, and C_12_), acid chain length (C_4_, C_8_ and C_12_), and reaction time (2, 4, and 6 h). Experiments were run following a random sequence. A further central point was added, corresponding to the combination of each independent variable taken at an intermediate level (70 °C; alcohol = C_8_; acid = C_8_; time point = 4 h). The response measured for each experiment was the conversion rate of the acid determined by GC-FID analysis.

### 2.8. Enzymatic Esterification Reactions

Reactions were performed using equimolar amounts (6.0 mmol) of carboxylic acid and alcohol and 2.5% of the immobilized enzyme (1% *w w*^−1^ enzyme/beads) calculated on the total amount of the monomers (2.5% *w w*^−1^ enzyme/total monomers weight). The reactions were carried out at different temperatures (50 °C, 70 °C, or 90 °C) using the multipoint reactor Carousel 12 plus reaction station purchased (Radleys, Saffron Walden, UK). Reactions were conducted in 50 mL reaction tubes continuously mixed using a magnetic stirrer (400 rpm). Afterwards, 10 µL of sample were taken at different timepoints (2, 4, and 6 h) and diluted in 10 mL of CHCl_3_; 10 µL of toluene were added as an internal standard. A blank of each reaction was performed without adding the biocatalyst. The samples were then analyzed via gas chromatography (GC-FID) as detailed in Table S3 in ESI. Calibration curves are shown in Figure S7 in ESI.

### 2.9. Gas Chromatography (GC-FID)

Each sample from the esterification reaction was diluted 1:4 (750 µL CHCl_3_ and 250 µL sample) with CHCl_3_ in 1.5 mL HPLC vials. Gas chromatography was performed in 45 min up to 250 °C, using CHCl_3_ as the washing solvent. Gas chromatography was carried out using an Agilent Technologies GC system (Agilent Technologies 6890N Network GC System, Santa Clara, CA, USA) connected to a J&W 122–3232 250 °C max DB-FFAP column with a capillary of 30.0 m × 250 μm × 0.25 μm nominal. The Injector (HO 6890 series) provided an H_2_ flow rate at 40 mL/min, airflow at 450 mL/min, and N_2_ makeup flow equal to 20.

### 2.10. Enzymatic Polycondensation Reactions

**Figure 1:**
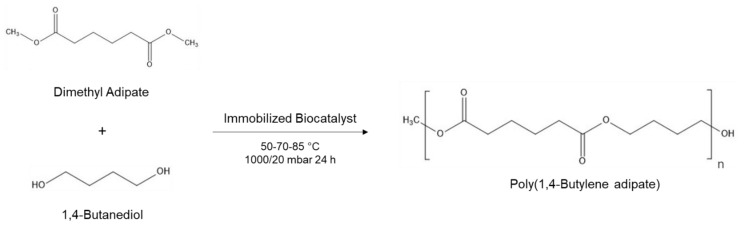
Example of enzymatic polycondensation reaction. Equimolar amounts of diesters and diols and 10% of the immobilized enzyme (1% *w w*^−1^ enzyme/beads) react at different temperatures at 1000 mbar for 6 h to form oligomers and then at 20 mbar for 18 h to combine and elongate the polymer chains. In the figure, for instance, dimethyl adipate and 1,4-butanediol catalyzed by the immobilized enzyme yield poly(1,4-butylene adipate).

Polycondensation reactions (Figure 1) were conducted as previously reported by Pellis et al. [25]. Equimolar amounts of diester and diol (0.006 mol) and 10% of the immobilized enzyme (1% *w w*^−1^ enzyme/beads) calculated on the total amount of the monomers (10% *w w*^−1^ enzyme/total monomers weight) were reacted at different temperatures at 1000 mbar for 6 h. The reactions were conducted in 25 mL round-bottom-flasks using a multipoint reactor Starfish multi-experiment workstation (Radleys, UK). After 6 h of reaction, reduced pressure was applied (20 mbar) by connecting the single reaction flasks to a Schlenk line connected to a vacuum pump V-300 (BÜCHI) connected to a pressure controller I-300 interface (BÜCHI). After 18 h (total reaction time: 24 h), the reaction mixture was recovered, dissolving the reaction products in Me-THF and removing the biocatalyst through a filtration step using a cotton filter. The solvent was then removed and the polymers were analyzed without further purification steps.

### 2.11. Proton Nuclear Magnetic Resonance (^1^H-NMR)

All ^1^H-NMR spectra were recorded on a Bruker Advance II 400 (resonance frequencies 400.13 MHz for ^1^H) equipped with a 5 mm observe broadband N_2_-cooled probe head (Prodigy) with z-gradients at room temperature with standard Bruker pulse programs. The samples were dissolved in 0.6 mL of CDCl_3_ (99.8% D). Chemical shifts are given in ppm, referenced to residual solvent signals (7.26 ppm for ^1^H). ^1^H-NMR data were collected with 32k complex data points and apodized with a Gaussian window function (lb = −0.3 Hz and gb = 0.3 Hz) prior to Fourier transformation. Signal-to-noise enhancement was achieved by multiplication of the FID with an exponential window function (lb = 1 Hz).

### 2.12. Gel Permeation Chromatography (GPC)

The samples were dissolved in CHCl_3_ at a concentration of 2 mg mL^−1^ and filtered through cotton filters. Gel permeation chromatography was carried out at 30 °C on an Agilent Technologies HPLC System (Agilent Technologies 1260 Infinity) connected to a 17,369 6.0 mm ID × 40 mm L HHR-H, 5 μm Guard column and a 18,055 7.8 mm ID × 300 mm L GMHHR-N, 5 μm TSK gel liquid chromatography column (Tosoh Bioscience, Tessenderlo, Belgium) using CHCl_3_ as an eluent (at a flow rate of 1 mL min^−1^). An Agilent Technologies G1362A refractive index detector was employed for detection. The molecular weights of the polymers were calculated using linear polystyrene calibration standards (250–70,000 Da) purchased from Sigma-Aldrich.

## 3. Results and Discussion

### 3.1. Enzyme Immobilization on Polypropylene Beads

Enzymes were immobilized on polypropylene beads using the protocol described in Section 2.3 using various *w/w* percentages of biocatalyst based on the amount of solid support. The used percentages were 1%, 2.5%, and 5% (*w/w*).

**Figure 2:**
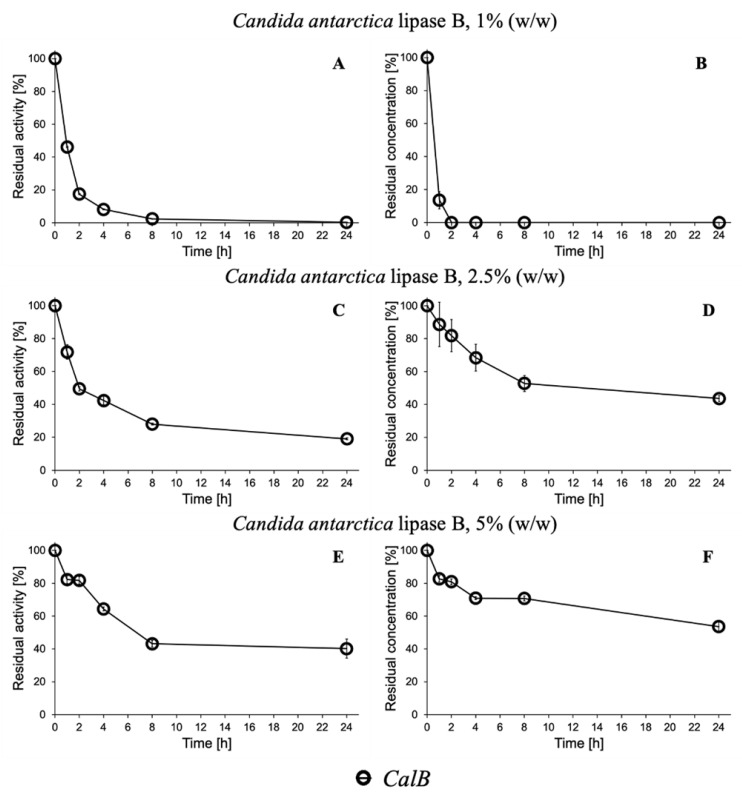
Immobilization of *Candida antarctica* lipase B (CaLB) onto Accurell MP 1000 (polypropylene) beads in 0.1 M Na_2_HPO_4_/NaH_2_PO_4_ buffer at pH 8 with different enzyme concentrations. Remaining para-nitrophenyl butyrate activity (**A**,**C**,**E**) and protein concentration (**B**,**D**,**F**) of the supernatant for CaLB concentrations of 1% (**A**,**B**), 2.5% (**C**,**D**), and 5% (**E**,**F**). The figure shows the mean ± SD.

The decrease of the activity due to the immobilization onto polypropylene beads is shown in residual activity of the supernatant (%), since every timepoint data was divided by the first timepoint result (0 h, starting point of the immobilization procedure). Results for the protein concentration determination are also presented in residual concentration (%) as explained above for the esterase activity assay. The immobilization results obtained using the lipase B from *Candida antarctica* (CaLB) are presented in Figure 2 as an example of the performed immobilization procedures.

After 8 h of immobilization CaLB at 1% *w/w*, <1% of both the residual activity and concentration was detected on the supernatant sample, indicating that >99% of the biocatalyst was successfully adsorbed to the solid support (Figure 2). When immobilized with higher concentrations of 2.5% and 5%, only a partial absorption of the enzyme was seen, with adsorption efficiencies of 81% and 63% for the 2.5% and 62% and 45% for the 5%, respectively (Figure 2). These results could be explained by the fact that polypropylene beads very saturated with protein at the lower enzyme concentration were applied.

Similar results, in which 1% of the formulations were completely adsorbed to the solid support after 8 h, were obtained as well for the other hydrolytic enzymes *Humicola insolens* cutinase (HiC), *Thermomyces lanuginosus* lipase (TLL), *Thermobifida cellulosilytica* cutinase 1 (Thc_Cut1), and cutinase 2 (Thc_Cut2) in their immobilization procedures (Figures S1–S3 in ESI).

In particular, our data achieved using CaLB 1% perfectly fit the trend of the remaining esterase activity presented in the work of Weinberger et al. [16], in which a similar immobilization procedure was used. In this work, after 1 and 24 h of reaction, 49% and almost all the enzyme was bound to the carrier, while 46% and >99% were accomplished in our work, showing a very similar adsorption pattern.

Furthermore, regarding concentrations 2.5% and 5% *w/w* applied, CaLB presented very similar results to HiC. In fact, after 24 h of reaction, both enzymes applied at 2.5% *w*/*w* showed about 80% immobilization (81% CaLB Figure 2C, 85% HiC Figure S1C) while using 5% preparations, and the same adsorption efficiency of 62% was obtained for both enzymes (CaLB Figure 2E, HiC Figure S1E).

A hypothesis that the rate of the immobilization to the polypropylene beads (hydrophobic support) was dependent on the superficial hydrophobicity of the enzyme was formulated. However, the GRAVY index (GRand AVerage of hYdropathicity index) calculated for each enzyme according to their superficial amino acids components available on PDB (Protein data bank) did not show any significant relation (Figure S4 in ESI).

Since the 1% (*w*/*w*) preparations showed the highest rate of enzyme successfully immobilized on the solid support, they were adopted for the subsequent reactions both for short-esters and polyesters synthesis.

### 3.2. Enzymatic Esterification Reactions

#### 3.2.1. Design of Experiments (DoE) Approach

After immobilization of the enzymes, the software MODDE^®^ Pro 13 (Sartorius) was used to develop a full-factorial design of experiments (DoE) to analyze thermostability and selectivity of the immobilized hydrolases towards alcohols and acids with different chain lengths in short-esters synthesis reactions. A model was constructed for each of the coefficients involved in the DoE (temperature, alcohol length, acid length, and reaction time) and the resulting reactions for verification were defined. The response measured for each experiment was the conversion rate of the acid (calculated via GC analysis). All the reactions performed for each enzyme are shown in Table S1 in ESI.

**Figure 3:**
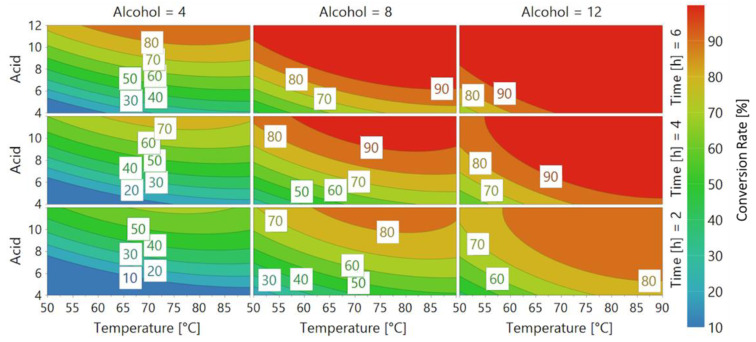
4D contour response of *Candida antarctica* lipase B (CaLB). Every graph shows a fixed alcohol chain length (Alcohol = 4, 8, 12 carbon atoms) at a different time (Time = 2, 4, 6), while Acid length and Temperature [°C] vary respectively on the Y and X axes. Different colors represent Conversion rate, as shown in the legend on the right.

After performing each experiment from the DoE, the data were compared to the model in a 4D contour response. The coefficients are shown in the axes while the response (the conversion rate) is represented by different colors in the plot (Figure 3).

#### 3.2.2. *Candida antarctica* Lipase B (CaLB) 4D Contour Response

The first part of the work was performed using CaLB, since this enzyme is undoubtedly one of the most widely studied biocatalysts in esterification reactions due to its extraordinary properties, such as the high catalytic activity and selectivity combined with a broad range of thermostability. Furthermore, its commercial availability (as a free and immobilized catalyst) has made CaLB one of the most applied enzymes in flavor-esters synthesis [26].

According to the literature [27,28], we tested the CaLB selectivity towards long-chain alcohols and acids. As it is visible in the top right graph in Figure 3, with the 12 carbon-chain alcohol the conversion rate was over 90% for each tested temperature, except for the 50–65 °C range, where a still very high 80% conversion rate was obtained. Regarding thermostability, we confirmed that CaLB is a thermostable enzyme in synthesis, showing the T_OPTIMUM_ in the 80–85 °C temperature range. In the same figure it is possible to observe that the conversion rates started to decrease at temperatures >85 °C. On the other hand, CaLB expressed limited conversion rates (<40%) towards small-chain alcohols and acids (4 and 6 carbon chain length) even after a reaction time of 6 h (Figure 3). These data are also confirmed by previous publications that used CaLB as the catalyst, where a decrease in activity above 85 °C in the polymerization of L-malic acid polyesters was reported by Yao et al. (2011) [27]. Furthermore, our results reflected very well the selectivity pattern reported by Pellis et al. (2018), in which CaLB showed its preference towards long-chain diols instead of short-chain compounds in polycondensation reactions [7].

In this first test, performed to validate our model using a well-known and characterized enzyme, the CaLB optimum temperature at 85 °C and its reduction in activity above this threshold was confirmed. Moreover, CaLB expressed the highest conversion rate (>90%) using lauric acid (acid = 12) reacted with 1-dodecanol (alcohol = 12), therefore leading to the conclusion that its selectivity is towards long-chain alcohols and acids.

#### 3.2.3. *Humicola insolens* Cutinase (HiC) 4D Contour Response

HiC is a widely studied cutinase due to its hydrolytic activity towards a variety of polyesters, including PET. However, only in the last decade this biocatalyst’s synthetic activity in polymerization reactions has been investigated [12,13].

**Figure 4:**
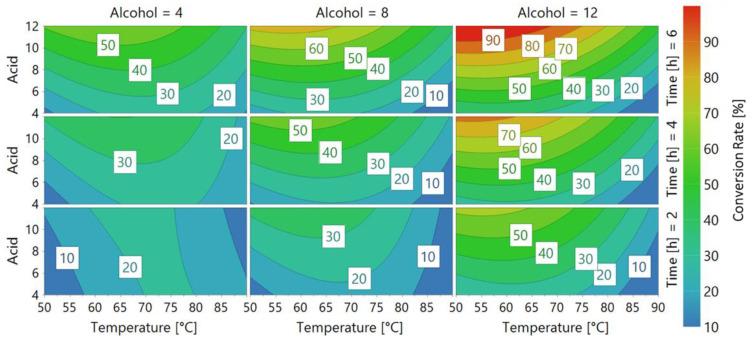
4D contour response of *Humicola insolens* cutinase (HiC). Every graph shows a fixed alcohol chain length (Alcohol = 4, 8, 12 carbon atoms) at a different time (Time = 2, 4, 6), while Acid length and Temperature [°C] vary respectively on the Y and X axes. Different colors represent Conversion rate, as shown in the legend on the right.

The 4D response contour obtained from the DoE performed using the HiC preparation showed both similarities and differences to CaLB preparation. Figure 4 clearly shows that similarly to CaLB, HiC’s selectivity was directed towards long-chain alcohols and acids. In fact, a 90% conversion rate was obtained after only 6 h of reaction when using the 12 carbon-chain alcohol. HiC showed a wide range of conversion rates related to the acid length, as is visible in the top left graph, where with small acid chains, only a 30% conversion rate was obtained. When using the 8 carbon-chain acid, a rate between 60% and 70% was achieved. Moreover, we confirmed that HiC is a thermostable enzyme that showed its T_OPTIMUM_ in synthesis at a temperature between 65–70 °C, while a large decrease in the conversion rate was observed above this temperature range. These data are also confirmed by previous publications using HiC as the catalyst where the same T_OPTIMUM_ at 70 °C and loss of activity above this value in lactone ring-opening and condensation polymerization reactions were reported by Hunsen et al. (2007) [12]. Furthermore, our observations fully agreed with the selectivity pattern reported by Feder and Gross (2010), in which HiC showed its high selectivity towards long-chain diols and diacids [22]. According to this trend, in our work, HiC showed meagre results with small chain alcohols and acids (acid = 4, 6), where only low conversion rates (<20% after 4 h, <30% after 6 h) were achieved.

We confirmed the optimum temperature of HiC at 70 °C and its consequent decrease of activity above this value. Moreover, similarly to CaLB, HiC exhibited the highest conversion rate (>90%) using lauric acid (acid = 12) when reacted with 1-dodecanol (alcohol = 12), therefore leading to the conclusion that its selectivity is towards long-chain alcohols and acids, while poor results were achieved using short-chain compounds.

#### 3.2.4. *Thermobifida cellulosilytica* Cutinase 1 (Thc_Cut1) 4D Contour Response

*Thermobifida cellulosilytica* cutinase 1 (Thc_Cut1) is a fairly recently developed enzyme, and its applications have yet to be explored, although excellent results have been obtained in the degradation of polyesters [29]. Regarding its synthetic activity, Thc_Cut1 has already proven its interesting polycondensation activity in previous studies [14,23]. Since it was the first time a complete optimization of this enzyme was carried out, a full-factorial design of experiment was planned.

**Figure 5:**
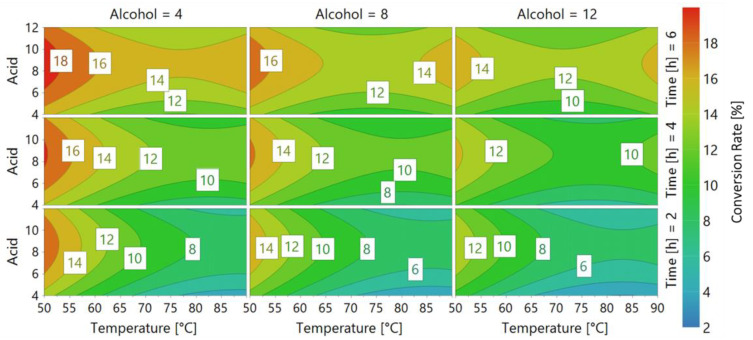
4D contour response of cutinase 1 from *Thermobifida cellulosilytica* (Thc_Cut1, using *Escherichia coli* as host). Every graph shows a fixed alcohol chain length (Alcohol = 4, 8, 12 carbon atoms) at a different time (Time = 2, 4, 6), while Acid length and Temperature [°C] vary respectively on the Y and X axes. Different colors represent Conversion rate, as shown in the legend on the right.

As shown in Figure 5, Thc_Cut1 exhibited a preference towards short-chain alcohols (C_4_) and medium-chain carboxylic acids (C_8_) as substrates in esterification reactions; the maximum conversion rate (18%) was reached at 50 °C, the temperature optimum for the enzyme. In fact, as the experimental temperature increased, the efficiency decreased to the minimum reached at 90 °C, when the enzyme was likely denatured.

Our results on temperature optimum were consistent with others previously published, indicating that, in polycondensation reactions, Thc_Cut1 is more efficient at 50 °C than at 70 °C, with C_4_ monomer conversion rates in 24 h of ~80% and ~37%, respectively [6,23].

Concerning the substrate specificity, our results were again consistent with previous works on Thc_Cut1-catalyzed polycondensation of dimethyl adipate with C_4_, C_6_, and C_8_ linear diols. These results indicated that Thc_Cut1 exhibited its highest activity towards C_4_ diols [6]. Interestingly, the enzyme preferred the C_4_ to C_8_ diol regardless of whether it was combined with C_6_, C_8_, or C_10_ diester monomers in reactions performed at 50 °C [23].

#### 3.2.5. *Thermomyces lanuginosus* Lipase (TLL) and Aspergillus Niger Lipase (AnL) 3D Surface Responses

TLL has emerged in the past years as one of the more versatile hydrolases for applications in industrial fields. Despite this, its utilization as catalyst in esterification reaction is not fully exploited [30]. On the other hand, *Aspergillus niger* is among the most well-known lipase producers and its enzyme is suitable for many uses in industrial applications [31,32]. Besides this, AnL synthesis activity in esterification reactions has not been extensively investigated [33].

**Figure 6:**
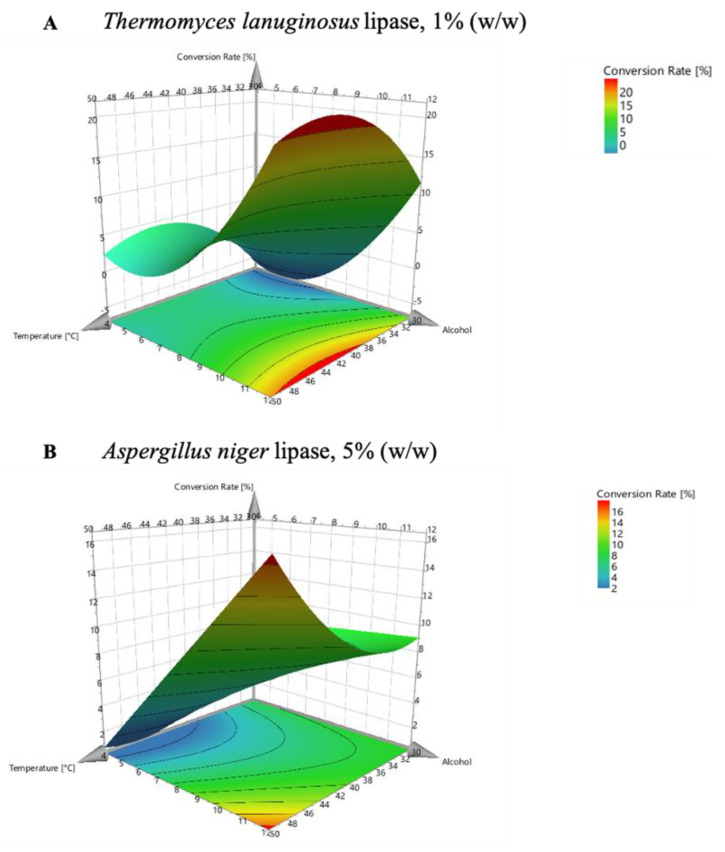
3D surface responses of *Thermomyces lanuginous* lipase (**A**) and *Aspergillus niger* lipase (**B**). Reactions with acid = 8 carbon atoms and time = 4 h are shown, while temperature [°C], alcohol length, and conversion rate (%) vary on the x, y, and z axes, respectively.

According to the literature, both TLL and AnL exhibit their activity only in a short range of temperatures. In particular, it was reported their rapid decrease or lack of activity over the 55–60 °C temperature range [30,34,35]. It was therefore necessary to adapt the previous DoE for these two enzymes: temperature range was changed so that enzymatic esterification reactions were performed at 30 °C, 40 °C, and 50 °C. Since this variation made it impossible to use lauric acid (melting point = 43.2 °C), the DoE performed for AnL and TLL was limited to a fractional-factorial design instead of the previous full-factorial design. As a consequence, the 4D response contour was not achievable. Instead, 3D surface responses are visualized in Figure 6.

Both enzymes showed a particularly limited conversion rate. In fact, TLL and AnL exhibited only 20% and 16% highest conversion rates with 8 carbon-chain acid and after 4 h of reaction, respectively, which are still very low if compared with the 90% (CaLB, Figure 3) and 60% (HiC, Figure 4) conversion rates. On the other hand, their activity was similar to the one showed by Thc_Cut1 (~18%). These data are also confirmed by previous publications using these enzymes as the catalysts, where poor esterification yield (<40%) with butyric acid and 1-butanol were obtained in absence of water after 24 h reaction using AnL, as reported by Verissìmo et al. (2015) [33]. Regarding TLL, Gumel et al. (2016) reported low conversion yield achieved in a continuous flow microreactor for methyl butanoate synthesis [30].

Both TLL and AnL showed low activity in the reactions performed (20% and 16%, respectively). For this reason, and for their inability to work at high temperatures (rapid decrease of activity above the 55–60 °C temperature range), which is a key point in the enzymatic synthesis field, TLL and AnL were not selected for carrying out the polycondensation reactions described in the following section. In fact, high catalytic activity and reaction temperature are necessary to perform enzymatic polycondensations, since the melting point of longer diols is frequently >50 °C. On the contrary, since its promising polycondensation activity has already been proved in previous studies [14,23], Thc_Cut1 was adopted for the subsequent polyesters synthesis, as well as CaLB and HiC.

### 3.3. Enzymatic Polycondensations

All the reactions were performed in bulk (i.e., solvent-less) in order to be as environmentally friendly as possible despite having to use a solvent for the work-up phase due to the necessity of removing the biocatalyst from the reaction mixture. The progression of the reaction was monitored via two techniques: ^1^H-NMR and GPC.

Using ^1^H-NMR, it was possible to calculate the monomer’s conversion monitoring both the intensity reduction of the signal at 3.7 ppm (-CH_2_-CH_2_-OH) in the ^1^H-NMR spectra of the polymerization products together with the intensification of the signal at 4.1 ppm (CH_2_-CH_2_-O-C=O). A second calculation was made based on the methanol that was released as by-product, therefore leading to the disappearance of the -OCH_3_ group of the diester at 3.7 ppm (Figure S5 in ESI). The integration of the peak at 4.1 ppm compared with the peak of the -CH_2_ adjacent to the carbonylic carbon group of the diester (2.3 ppm) was used to determine the monomer conversion rate, since this signal is constant and does not change with the progression of the reaction. Other important polymers properties, such as weight average molecular weight (M_w_), average number molecular weight (M_n_), dispersity (Đ), and degree of polymerization (DP), were obtained from the GPC analysis and provided information on the chain length distribution of the various obtained polyesters.

**Figure 7:**
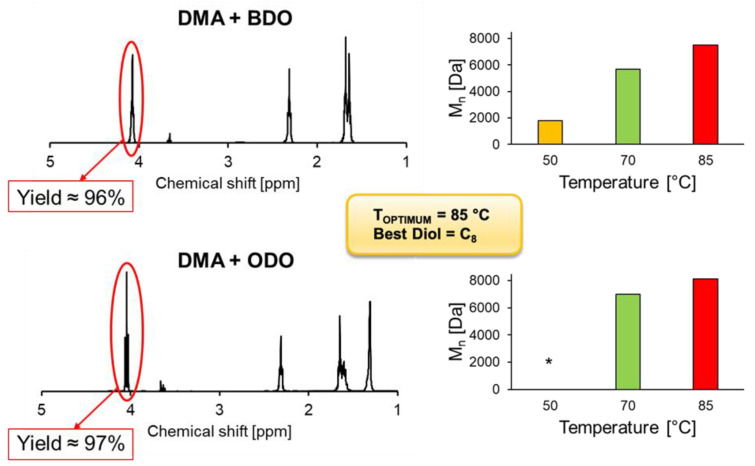
Polycondensation of DMA with 1,4-BDO and 1,8-ODO catalyzed by 1% (*w*/*w*) of immobilized CaLB after 24 h of reaction. On the left, ^1^H-NMR calculations of DMA reacted with BDO and ODO at 85 °C. Calculations were performed by comparing the ration between the signal methylene groups adjacent to -OH of BDO/ODO and the methylene groups of DMA (assumed as constant). On the right, the graphs show the average number of molecular weight (M_n_) accomplished at different temperatures [50, 70, 85 °C]. M_n_ was calculated via GPC calibrated with low molecular weight polystyrene standards 250–70,000 Da. * Reaction at 50 °C using ODO was not carried out due to the impossibility of obtaining a homogeneous melt (melting point of ODO ~61 °C).

Following the results on short esters obtained with the DoE (Section 3.2), the temperature optimum of the CaLB preparation was tested, also regarding the synthesis of aliphatic polyesters. Polycondensation reactions using dimethyl adipate (DMA) and the diols 1,4-butanediol (BDO) and 1,8-octanediol (ODO) were performed. As it is possible to observe from the GPC data in Figure 7, CaLB showed its T_OPTIMUM_ in synthesis at 85 °C. In fact, The M_n_ of 7500 Da obtained using BDO at this temperature was higher than the M_n_ of 5700 Da at 70 °C and 1800 Da at 50 °C. Furthermore, our CaLB immobilized on polypropylene beads showed better results at 85 °C than the commercial one immobilized on macroporous acrylic resin beads Novozym 435 (M_n_ = 4300 Da), shedding light on promising prospects for the future (Table S2 in ESI).

Moreover, as it is reported on the ^1^H-NMR graphs in Figure 7, we verified, according to the literature [7,36], that CaLB exhibits its selectivity towards long-chain diols, as well as long-chain alcohols and acids previously proven (Section 3.2.2). Actually, as was also noticed by Pellis et al. (2018), CaLB led to similar conversions with BDO, HDO (1,6-hexandiol), and ODO when they were used as diols. In fact, an interesting M_n_ of 8100 Da with a monomer conversion rate of 97% was achieved using ODO as the diol, while with BDO a similar conversion rate of 96% was achieved. Besides this, here as well, the M_n_ was higher than the one from iCaLB (M_n_ = 5000 Da).

**Figure 8:**
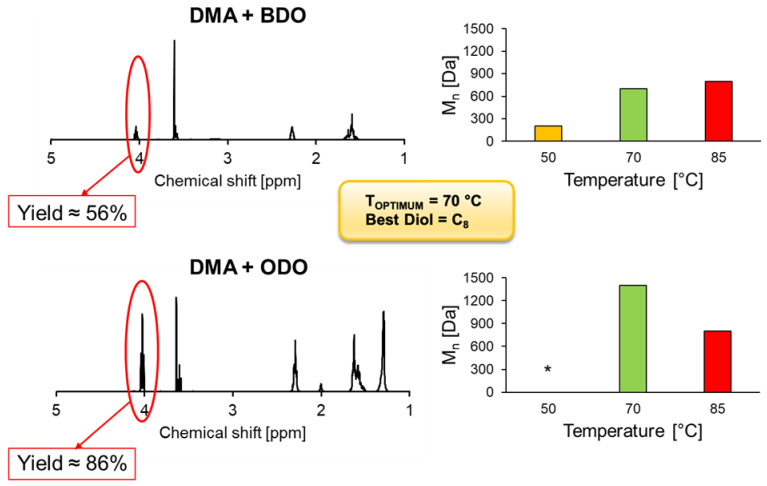
Polycondensation of DMA with 1,4-BDO and 1,8-ODO catalyzed by 1% (*w*/*w*) of immobilized HiC after 24 h of reaction. On the left, ^1^H-NMR calculations of DMA reacted with BDO and ODO at 70 °C. Calculations were performed by comparing the ration between the signal methylene groups adjacent to -OH of BDO/ODO and the methylene groups of DMA (assumed as constant). On the right, the graphs show the average number of molecular weight (M_n_) accomplished at different temperatures [50, 70, 85 °C]. M_n_ was calculated via GPC calibrated with low molecular weight polystyrene standards 250–70,000 Da. * Reaction at 50 °C using ODO was not carried out due to the impossibility of obtaining a homogeneous melt (melting point of ODO ~61 °C).

Carrying out the same reactions using HiC, we instead observed that the T_OPTIMUM_ proved to be at 70 °C (Figure 8). In fact, both the monomer conversion rate and the M_n_ were higher at 70 °C than other temperatures, using both BDO (56% and 700 Da at 70 °C than 52% and 200 Da at 50 °C) and ODO (86% and 1400 Da at 70 °C than 31% and 800 Da at 85 °C) as diols. Actually, at 85 °C using DMA and BDO, M_n_ resulted slightly higher than at 70 °C (800 Da instead of 700 Da), but the conversion rate clearly confirmed the T_OPTIMUM_ at 70 °C (56% instead of 7% at 85 °C). Moreover, the highest monomer conversion rate was obtained using ODO as diol at T_OPTIMUM_ (86% instead of 56% with BDO), showing HiC selectivity towards long-chain diols, as previously observed from the HiC DoE results and also as previously reported in the literature by Feder and Gross (2010), who observed HiC preference towards long-chain carbon diols (C_8_ and C_6_ instead of C_3_, C_4_ and C_5_) in copolymerization of sebacic acid [22]. Despite the high monomer conversion rate, polymers obtained using HiC presented lower molecular weights if compared with the ones synthesized using both CaLB formulations. In particular, the M_n_ < 2 kDa led to polymers that were not even solid at room temperature, but presented as viscous liquids. Upon functionalization, these short oligomers could have some interesting applications in personal and home-care products.

After using these two hydrolytic commercial enzymes, we performed the same polycondensation reactions using cutinase 1 from *Thermobifida cellulosilytica* (Thc_Cut1) produced using *E. coli* as a host and immobilized on polypropylene beads.

**Figure 9:**
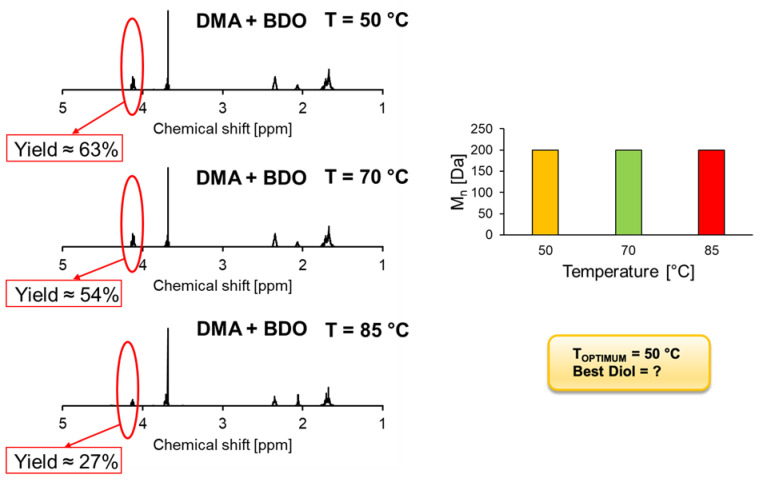
Polycondensation of DMA with 1,4-BDO catalyzed by 1% (*w*/*w*) of immobilized Thc_Cut1 after 24 h reaction. On the left, ^1^H-NMR calculations of DMA reacted with BDO at 50 °C, 70 °C and 85 °C. Calculations were performed by comparing the ration between the signal methylene groups adjacent to -OH of BDO and the methylene groups of DMA (assumed as constant). On the right, the graph shows the average number of molecular weight (M_n_) accomplished at different temperatures [50, 70, 85 °C]. M_n_ was calculated via GPC calibrated with low molecular weight polystyrene standards 250–70,000 Da.

Observing the GPC data (Figure 9), we could not identify at which temperature Thc_Cut1 expressed its T_OPTIMUM_, since M_n_ of 200 Da was achieved for every temperature tested. Analyzing the ^1^H-NMR results, it was possible to observe that the highest monomer conversion rate of 63% was obtained at 50 °C. With the increasing of the temperature, monomer conversion rate started to decrease, obtaining 54% and 27% at 70 °C and 85 °C, respectively, showing reduction of Thc_Cut1 activity. Hence, according to the literature [14], we verified Thc_Cut1 T_OPTIMUM_ at 50 °C. Since the ODO melting point is at around 61 °C, it was not possible to perform polycondensation reactions with this compound at 50 °C to determine Thc_Cut1 selectivity towards diols. However, interesting results in the literature regarding synthesis can already be found, underlining its preference towards small-chain carbon diols [6,14].

Further polycondensation reactions were performed at enzymes T_OPTIMUM_ using different building blocks, such as dimethyl sebacate (DMSe), which presents in its structure as an interesting long-linear carbon chain, and dimethyl itaconate (DMI), which shows an unsaturated double carbon bond in its structure, capable of creating a steric hindrance towards the active site of the enzymes [37].

Polycondensation reactions performed using DMSe (Table 1) led to promising results, confirming CaLB and HiC selectivity towards long carbon-chain compounds. In particular, polymers obtained using immobilized CaLB on polypropylene beads showed surprising M_n_ (7500 and 5100 Da), M_w_ (18,200 and 17,000 Da), and DP (~30 and ~16), and similar and even higher than commercial iCaLB (M_n_ 4000 and 5400 Da, M_w_ 13,500 and 13,800 and DP = 15 and 17 with BDO and ODO as diols, respectively). On the other hand, HiC showed the highest M_n_ obtained with BDO and ODO among all the different building blocks involved in the reactions (1000 and 3200 Da with BDO and ODO, respectively). Despite this, only 54% of the monomer conversion rate was achieved with BDO.

Polymers based on DMSe obtained using CaLB showed M_n_ lower than the ones from DMA (7500 and 5100 Da from DMSe instead of 8100 and 7500 Da from DMA). These results fully agreed with the observation made by Pellis et al. (2018), i.e., the adipic acid dimethyl esters lead to higher molecular weight products than the sebacic dimethyl esters when CaLB is applied as catalyst [7]. On the contrary, HiC revealed polymers with higher M_n_ using DMSe (3200 and 1000 Da) than DMA (1400 and 700 Da). This very well reflects the results achieved by Feder and Gross (2010), where polycondensations of different linear diacids and various diols using HiC immobilized on Amberzyme oxirane (AO) resin at 70 °C were performed, leading to the conclusion that HiC expresses its preference towards DMSe [22].

Using DMI as the diester (Table 2), independently from the diol used, only polyesters having limited M_w_ and M_n_ were obtained using both CaLB and HiC. This low reactivity of DMI was probably due to the steric hindrance produced by the unsaturated double-carbon bond and to the lower electrophilicity of the acyl carbon adjacent to the vinyl group [38]. In fact, molecular weights of only 900 and 600 Da of M_n_ were achieved using BDO with CaLB and HiC, respectively, while 700 Da of M_n_ was obtained using ODO with both these enzymes. Monomer conversion rate, in particular, reflects the problematic interaction, showing only 44% and 8% using BDO, while 62% and 36% were accomplished using ODO with CaLB and HiC, respectively. Moreover, DP perfectly underlined these enzymes’ difficulties towards DMI, since only meagre results were accomplished (DP < 5 using BDO and ODO with both CaLB and HiC). In no case, the polymers achieved showed results comparable to the ones obtained with the commercial iCaLB (M_n_ 2400 and 4100 Da, both 95% conversion rate and DP = 13 and 17). Our results fully agreed with the observation made by Barrett et al. (2010), i.e., CaLB-catalyzed polycondensation of itaconic acid derivates suffer from slow reaction kinetics caused by the poor reactivity of the acyl group, which undergoes the stabilizing resonance effect of the conjugated double-carbon bond [37]. This condition was widely investigated by Corici et al. (2015) who, using CaLB, shed light on a number of factors affecting the enzymatic polycondensation of DMI. Among them, it was demonstrated that not only the BDO concentration must be maintained low throughout the process, but its structure is not favourable for the elongation. Furthermore, optimal mass transfer and a homogeneous dispersion of the enzyme in the reaction mixture are amongst the most important conditions needed for achieving a reasonable elongation of the oligomers [38].

## 4. Conclusions

Different hydrolytic enzymes were successfully immobilized onto Accurell MP 1000 (polypropylene) beads. In order to study their thermostability and selectivity in synthesis reactions, MODDE^®^ Pro 13 (Sartorius) was adopted as software to develop a full-factorial design of experiment (DoE), in which enzyme transesterification activity to obtain flavor esters was investigated. Results showed that both *Candida antarctica* lipase B (CaLB) and *Humicola insolens* cutinase (HiC) expressed their selectivity towards long-chain alcohols and acids, while their optimum temperature were found at 85 °C and 70 °C, respectively. Further immobilized enzymes, such as *Thermomyces lanuginous* lipase (TLL) and *Aspergillus niger* lipase (AnL), were studied and found as unsuitable for subsequent polycondensations reactions due to their low thermostability and activity. On the contrary, *Thermobifida cellulosilytica* cutinase 1 (Thc_Cut1, expressed using *Escherichia coli* as the host) showed its preference towards short-chain alcohols and acids, indicating its optimum temperature at 50 °C. Dimethyl adipate (DMA) and two different diols, 1,4-butanediol (BDO) and 1,8-octanediol (ODO), were used as building blocks for biocatalyzed synthesis of polyesters through polycondensation reactions, using the previously characterized immobilized hydrolytic enzymes. For both CaLB and HiC, optimum temperature and selectivity towards long-chain compounds were confirmed. In particular, using CaLB, particularly interesting polyesters with DMA were achieved, showing high monomer conversion rates (>90%), average molecular weight (M_w_), and average number of molecular weight (M_n_) even higher than commercial CaLB immobilized on macroporous acrylic resin beads (M_n_ 7500 instead of 4300 Da using BDO and 8100 instead of 5000 Da using ODO). On the other hand, despite the high monomer conversion rate (>55%), polymers obtained using HiC presented lower molecular weights when compared with the ones accomplished using both CaLB formulations. Polycondensation reactions performed using Thc_Cut1 lead to meagre results as well, obtaining polyesters with M_n_ < 1 kDa. However, we confirmed this enzyme temperature optimum at 50 °C, since the conversion rate appeared to be the highest when compared with the ones at 70 °C and at 85 °C (63% instead of 54% and 27%, respectively). Afterwards, different building blocks were adopted for further polycondensation reactions using the commercial biocatalysts. Both CaLB and HiC expressed difficulties in synthesis towards dimethyl itaconate (DMI), revealing that its double-carbon bond creates steric hindrance towards enzymes active sites; while using dimethyl sebacate (DMSe), surprising results were achieved (18 kDa and 6 kDa), confirming these enzymes’ preferences towards long-linear carbon chains. In conclusion, we shed light on different hydrolytic enzyme properties in synthesis activity, providing a clear idea about how promising biocatalyzed synthesis of polyesters could become in the future. Our study contributed to show, even for the industrial scale, how enzymes could produce important economic advantages, such as milder reaction conditions (temperature and pressure) with consequent energy saving, environmentally friendly processes (since toxic catalysts and solvents are avoided), and selectivity towards bio-based monomers that otherwise could give side reactions and, hence, low conversion rates (such as itaconic acid and derivates)

## Figures and Tables

**Table 1:** Polycondensation of DMSe with 1,4-BDO and 1,8-ODO catalyzed by 1% (*w*/*w*) of different immobilized enzymes at their T_OPTIMUM_ after 24 h of reaction.

Diol	Imm. Enzyme	Temp. [°C]	M_n_ ^a^	M_w_ ^a^	Đ ^a^	M_o_	DP ^c^	Conv. (%) ^b^
1,4-butanediol	CaLB L4777	85	4000	13,500	3.43	256.3	15	93
	CaLB		7500	18,200	2.46		29	93
	HiC	70	1000	1300	1.20		4	54
1,8-octanediol	CaLB L4777	85	5400	13,800	2.56	312.4	17	91
	CaLB		5100	17,000	3.40		16	96
	HiC	70	3200	6000	1.88		10	91

^a^ Calculated via GPC calibrated with low molecular weight polystyrene standards 250–70,000 Da. ^b^ Calculated via ^1^H-NMR by comparing the ration between the signal methylene groups adjacent to -OH of BDO and the methylene groups of DMSe (assumed as constant). ^c^ Degree of polymerization (DP) = M_n_/molecular weight of the repeating unit (M_o_).

**Table 2:** Polycondensation of DMI with 1,4-BDO and 1,8-ODO catalyzed by 1% (*w*/*w*) of different immobilized enzymes at their T_OPTIMUM_ after 24 h of reaction.

Diol	Imm. Enzyme	Temp. [°C]	M_n_ ^a^	M_w_ ^a^	Đ ^a^	M_o_	DP ^c^	Conv. (%) ^b^
1,4-butanediol	CaLB L4777	85	2400	4500	1.90	184.2	13	95
	CaLB		900	1000	1.16		5	44
	HiC	70	600	600	1.00		3	8
1,8-octanediol	CaLB L4777	85	4100	9200	2.24	240.3	17	95
	CaLB		700	800	1.06		3	62
	HiC	70	700	800	1.07		3	36

^a^ Calculated via GPC calibrated with low molecular weight polystyrene standards 250–70,000 Da. ^b^ Calculated via ^1^H-NMR by comparing the ration between the signal methylene groups adjacent to -OH of BDO and the methylene groups of DMSe (assumed as constant). ^c^ Degree of polymerization (DP) = M_n_/molecular weight of the repeating unit (M_o_).

## Data Availability

All data not present in the main text or the ESI are available from the authors upon request.

## Supplementary Materials

The following are available online at https://www.mdpi.com/article/10.3390/ijms22168493/s1.
