# Supplementary material for: Biocatalyzed Synthesis of Flavor Esters and Polyesters: A Design of Experiments (DoE) Approach
Source: Int J Mol Sci. 2021 Aug 6;22(16):8493. doi: 10.3390/ijms22168493 (PMC8395215; doi:10.3390/ijms22168493)
Supplement: Supplementary file 1 [file ijms-22-08493-s001.zip › ijms-1308422-supplementary.pdf]

Electronic Supplementary Information

Biocatalyzed synthesis of flavor esters and polyesters using hydrolytic enzymes: a design of experiments (DoE) approach

Filippo Fabbri[1], Federico A. Bertolini[1], Georg M. Guebitz[1,2], Alessandro Pellis[1,*]

[1] University of Natural Resources and Life Sciences, Vienna, Department of Agrobiotechnology, Institute of Environmental Biotechnology, Konrad Lorenz Strasse 20, 3430, Tulln an der Donau, Austria

[2] Austrian Centre of Industrial Biotechnology, Konrad Lorenz Strasse 20, 3430, Tulln an der Donau, Austria

*Correspondence: alessandro.pellis@boku.ac.at

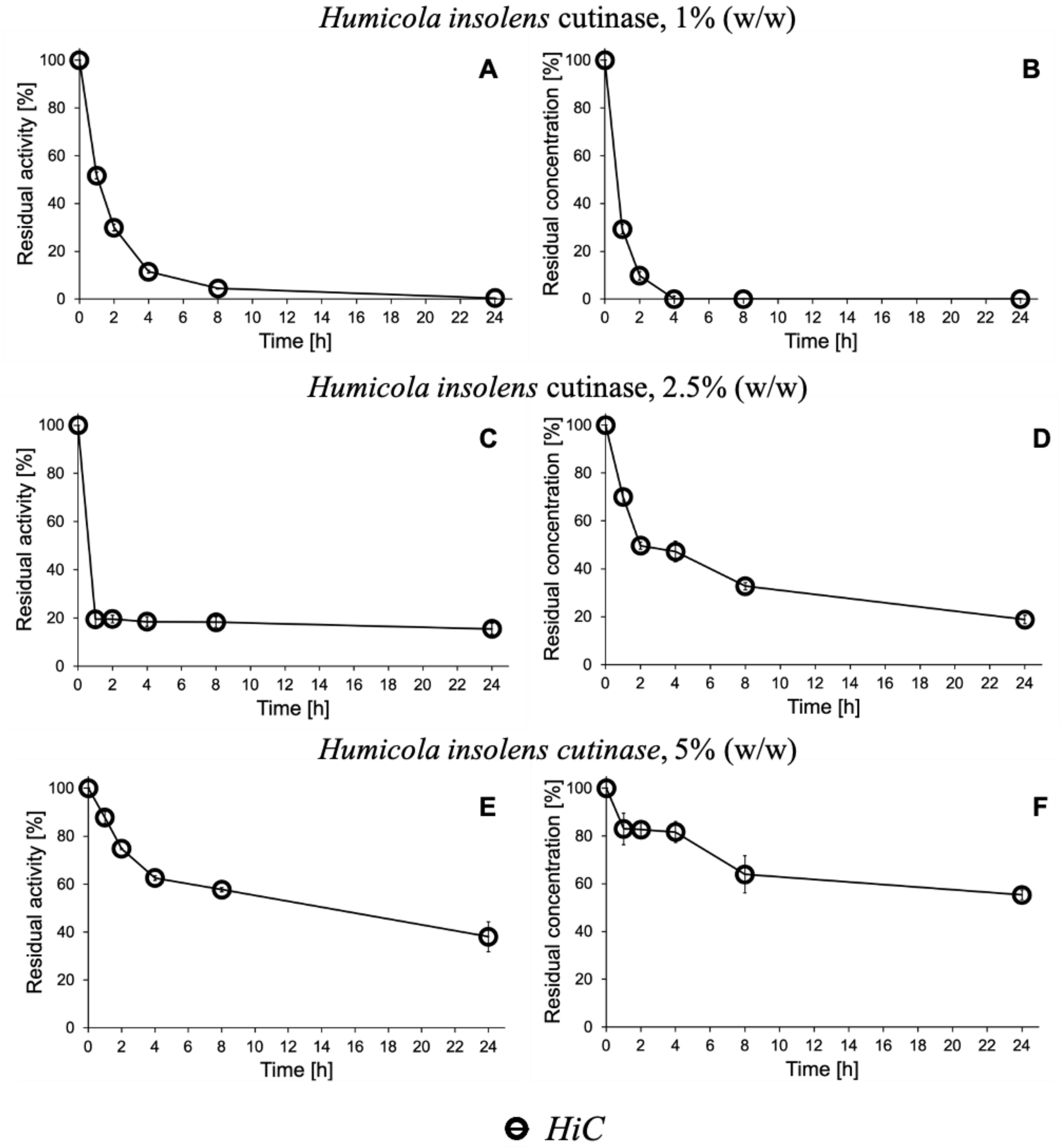

Figure S1. Immobilizations of *Humicola insolens* cutinase (HiC) onto Accurell MP 1000 (polypropylene) beads in 0.1 M Na_2HPO_4/NaH_2PO_4 buffer at pH 8 with different enzymes concentrations. Remaining para-nitrophenyl butyrate activity (A, C and E) and protein concentration (B, D and F) of the supernatant for HiC concentrations of 1% (A and B), 2.5% (C and D) and 5% (E and F). The figure shows the mean ± SD.

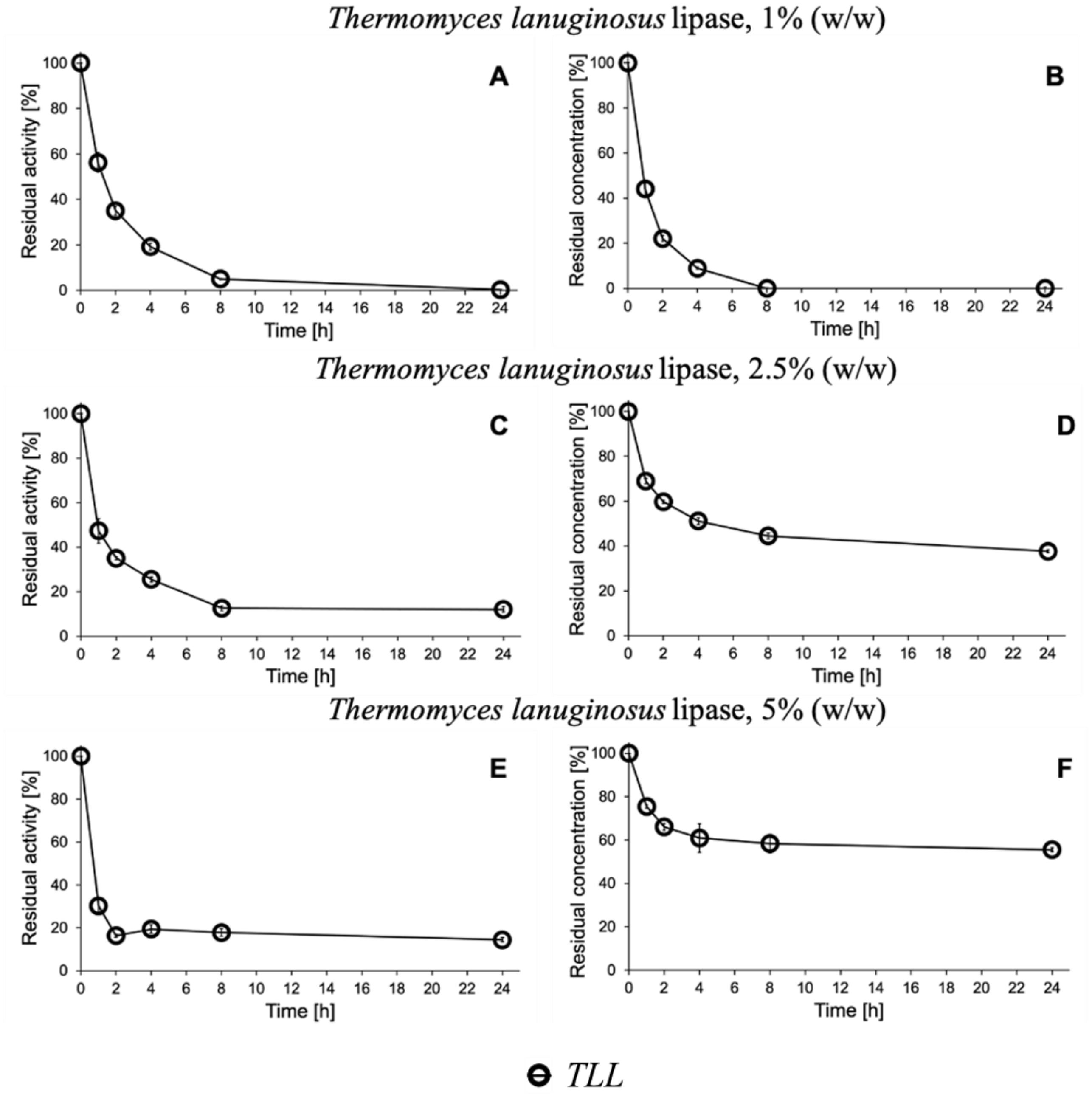

Figure S2. Immobilizations of *Thermomyces lanuginosus* lipase (TLL) onto Accurell MP 1000 (polypropylene) beads in 0.1 M Na_2HPO_4/NaH_2PO_4 buffer at pH 8 with different enzymes concentrations. Remaining para-nitrophenyl butyrate activity (A, C and E) and protein concentration (B, D and F) of the supernatant for TLL concentrations of 1% (A and B), 2.5% (C and D) and 5% (E and F). The figure shows the mean ± SD.

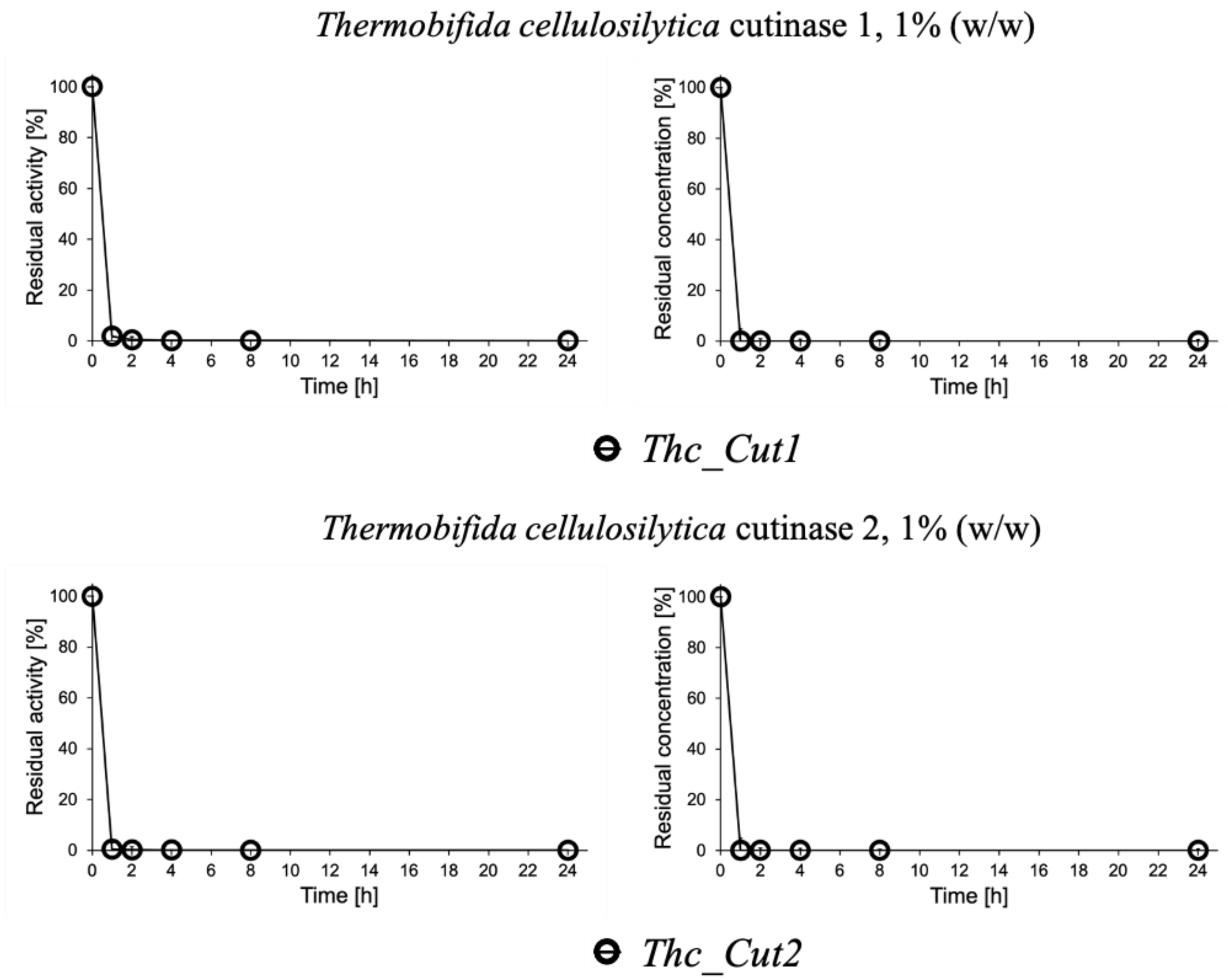

Figure S3. Immobilizations of Thc_Cut1 (above) and Thc_Cut2 (below) onto Accurell MP 1000 (polypropylene) beads in 0.1 M Na_2HPO_4/NaH_2PO_4 buffer at pH 8 with enzymes concentrations 1% (w/w), remaining para-nitrophenyl butyrate activity (on the left) and protein concentration (on the right) in the supernatant. The figure shows the mean ± SD.

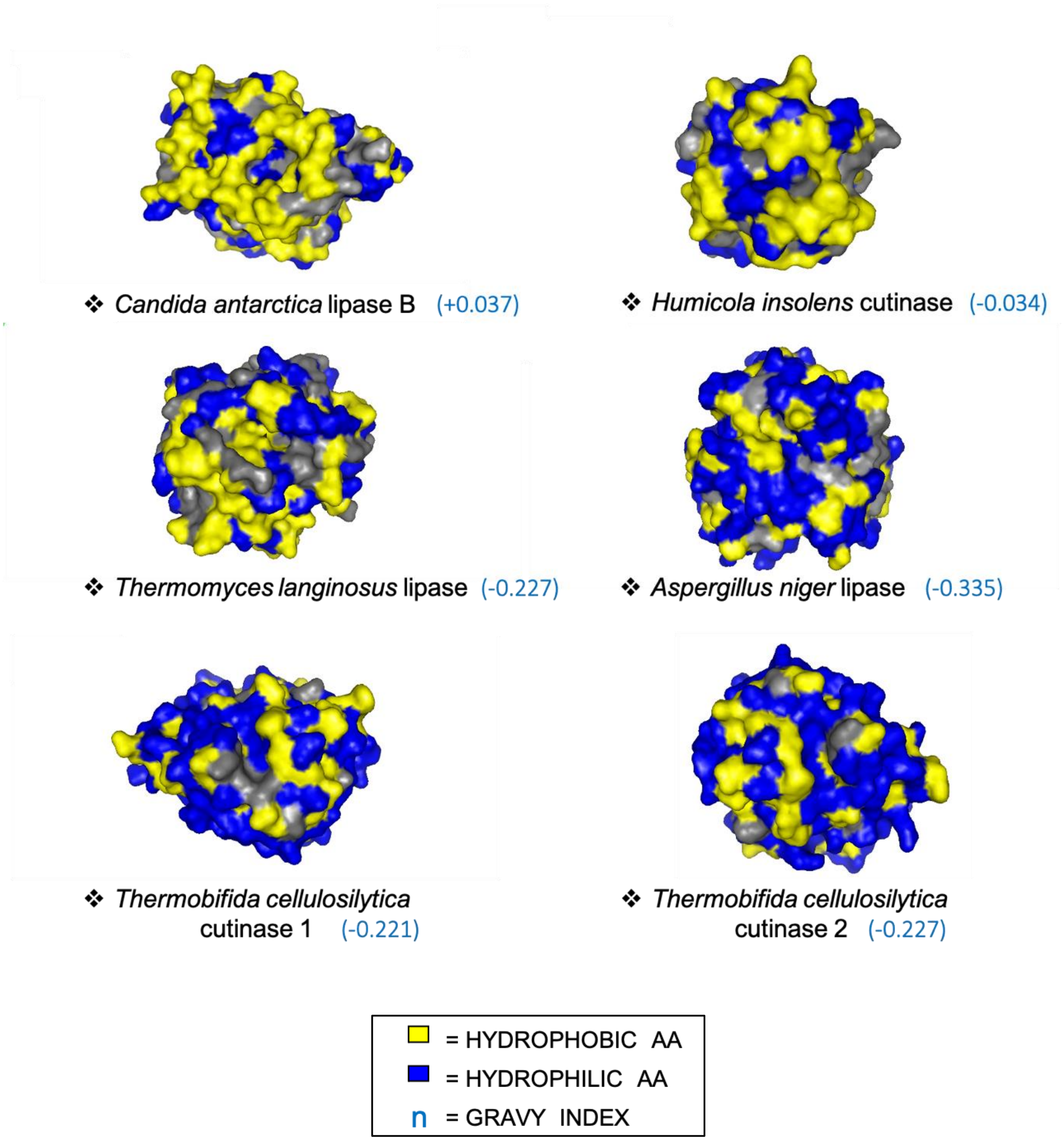

Figure S4. Gravy index calculations of different hydrolytic enzymes to verify if the rate of immobilization onto polypropylene beads (hydrophobic support) was proportional to the hydrophobicity of the enzymes. Higher the number of the gravy index, higher the hydrophobicity. Pictures were taken using Pymol while Gravy index was calculated using PDB (Protein DataBank) tools. PDB code for enzymes: CaLB (1tca), HiC (4oyy), AnL (1uwc), TLL (1du4), Thc_Cut 1 (5lui), Thc_Cut 2(5luj).

Table S1. The reactions involved in the full factorial design. Coefficient shown are temperature (50 °C, 70 °C and 90 °C), alcohol length (C_4, C_8 and C_{12}) and acid length (C_4, C_8 and C_{12}). Time points were withdrawn at different time (2, 4 and 6 hours). The last three reactions represent the central point of the DoE.

Reaction no°	Temperature [°C]	Acid length [C no°]	Alcohol length [C no°]
1	50	4	4
2	70	4	4
3	90	4	4
4	50	8	4
5	70	8	4
6	90	8	4
7	50	12	4
8	70	12	4
9	90	12	4
10	50	4	8
11	70	4	8
12	90	4	8
13	50	8	8
14	70	8	8
15	90	8	8
16	50	12	8
17	70	12	8
18	90	12	8
19	50	4	12
20	70	4	12
21	90	4	12
22	50	8	12
23	70	8	12
24	90	8	12
25	50	12	12
26	70	12	12
27	90	12	12
28	70	8	8
29	70	8	8
30	70	8	8

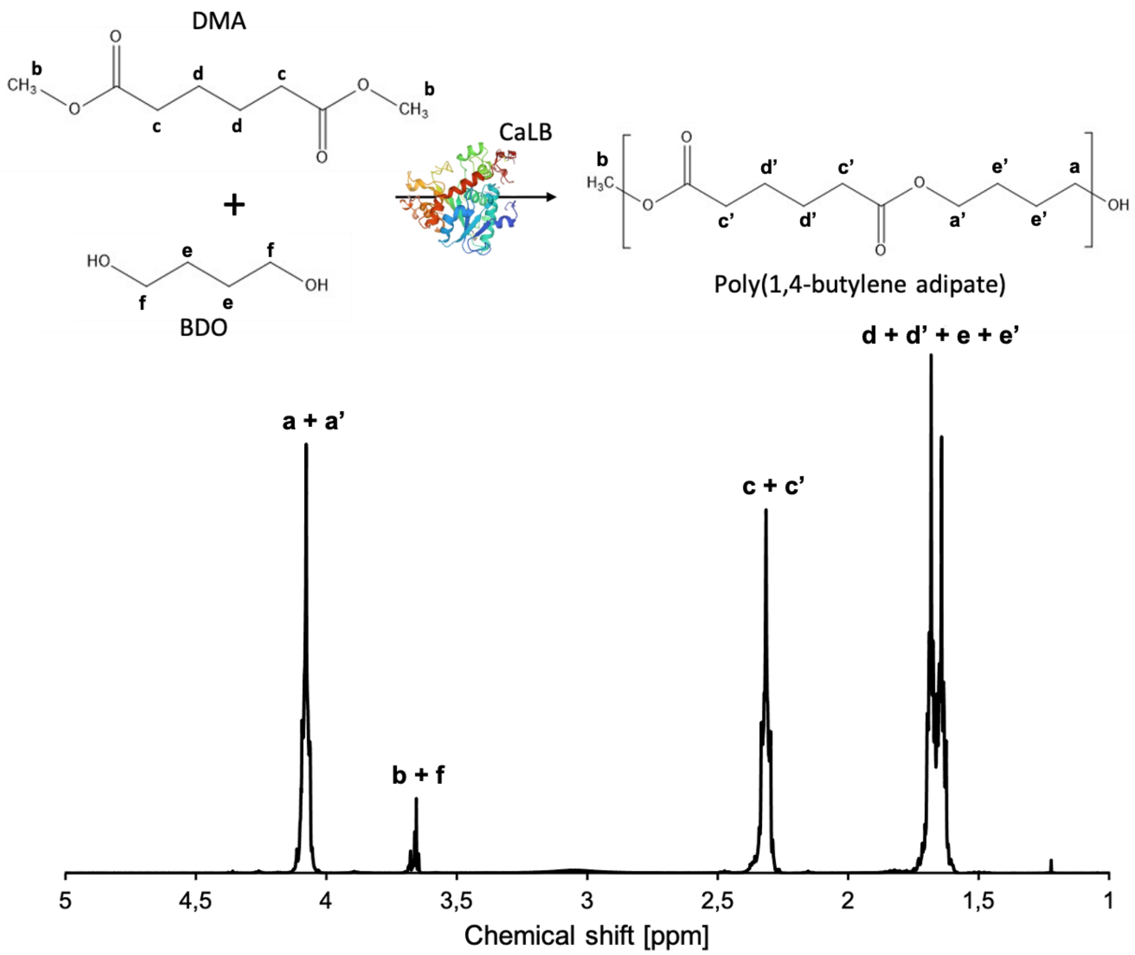

Figure S5. Detail of the ^{1}H-NMR spectrum of poly(1,4-butylene adipate) synthesized using immobilized CaLB starting from dimethyl adipate and 1,4-butanediol. Spectrum was recorded using $CDCl_3$ as solvent.

Table S2. Polycondensation of DMA with 1,4-BDO and 1,8-ODO catalysed by 10% (w/w) of different immobilized enzymes at their $T_{OPTIMUM}$ after 24h reaction.

Diol	Imm. enzyme	Temp.	M_n [a]	M_w [a]	Đ [a]	M_o	DP [c]	Conv. [%] [b]
1,4-butanediol	CaLB L4777	85°C	4300	14400	3.36	200.2	21.5	93
1,4-butanediol	CaLB	85°C	7500	19500	2.58		37.4	96
1,4-butanediol	CaLB	70°C	5700	11400	2.01		28.5	96
1,4-butanediol	CaLB	50°C	1800	4800	2.66		9.0	92
1,4-butanediol	HiC	85°C	800	900	1.12		4.0	7
1,4-butanediol	HiC	70°C	700	700	1.03		3.5	56
1,4-butanediol	HiC	50°C	200	400	1.71		1.0	52
1,4-butanediol	Thc_Cut1	85°C	200	300	1.53		1.0	27

1,4-butanediol	Thc_Cut1	70°C	200	300	1.59		1.0	54
1,4-butanediol	Thc_Cut1	50°C	200	400	1.96		1.0	63
1,8-octanediol	CaLB L4777	85°C	5000	22200	4.45	256.3	19.5	96
1,8-octanediol	CaLB	85°C	8100	22200	2.73		31.6	97
1,8-octanediol	CaLB	70°C	7000	16700	2.38		27.3	96
1,8-octanediol	HiC	85°C	800	800	1.10		3.1	31
1,8-octanediol	HiC	70°C	1400	2000	1.42		5.5	86

[a]Calculated via GPC calibrated with low molecular weight polystyrene standards 250-70 000 Da.

[b]Calculated via ^{1}H-NMR by comparing the ration between the signal methylene groups adjacent to -OH of BDO and the methylene groups of DMSe (assumed as constant).

[c]Degree of polymerization (DP) = M_n/molecular weight of the repeating unit (M_o).

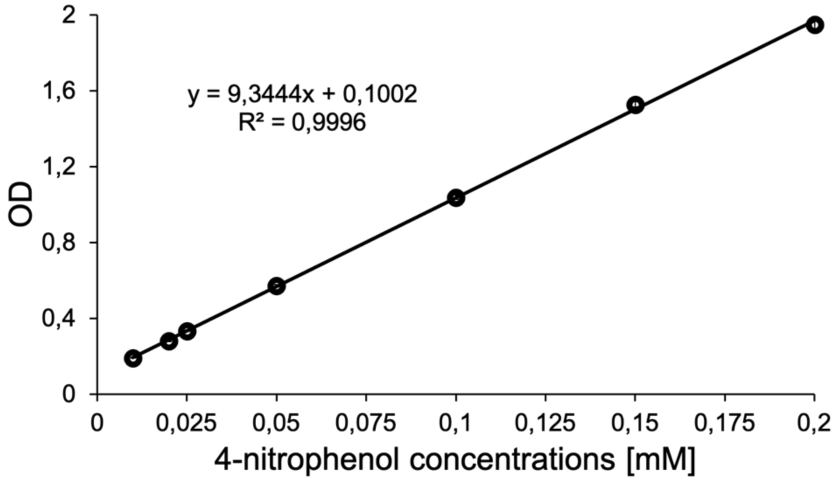

Figure S6. Determination of the extinction coefficient of 4-nitrophenol in 0.1 M Na_2HPO_4/NaH_2PO_4 buffer at pH 8.0. The figure shows the mean ± SD.

Table S3. Gas chromatography (GC-FID) parameters

**GC**	**C°/min**	**Next C°**	**Hold min**	**Run time**
Initial		50	5.00	5.00
Ramp 1	5.00	230	2.00	43.00
Ramp 2	25.00	250	2.00	45.80
Post-run		50	0.00	45.80

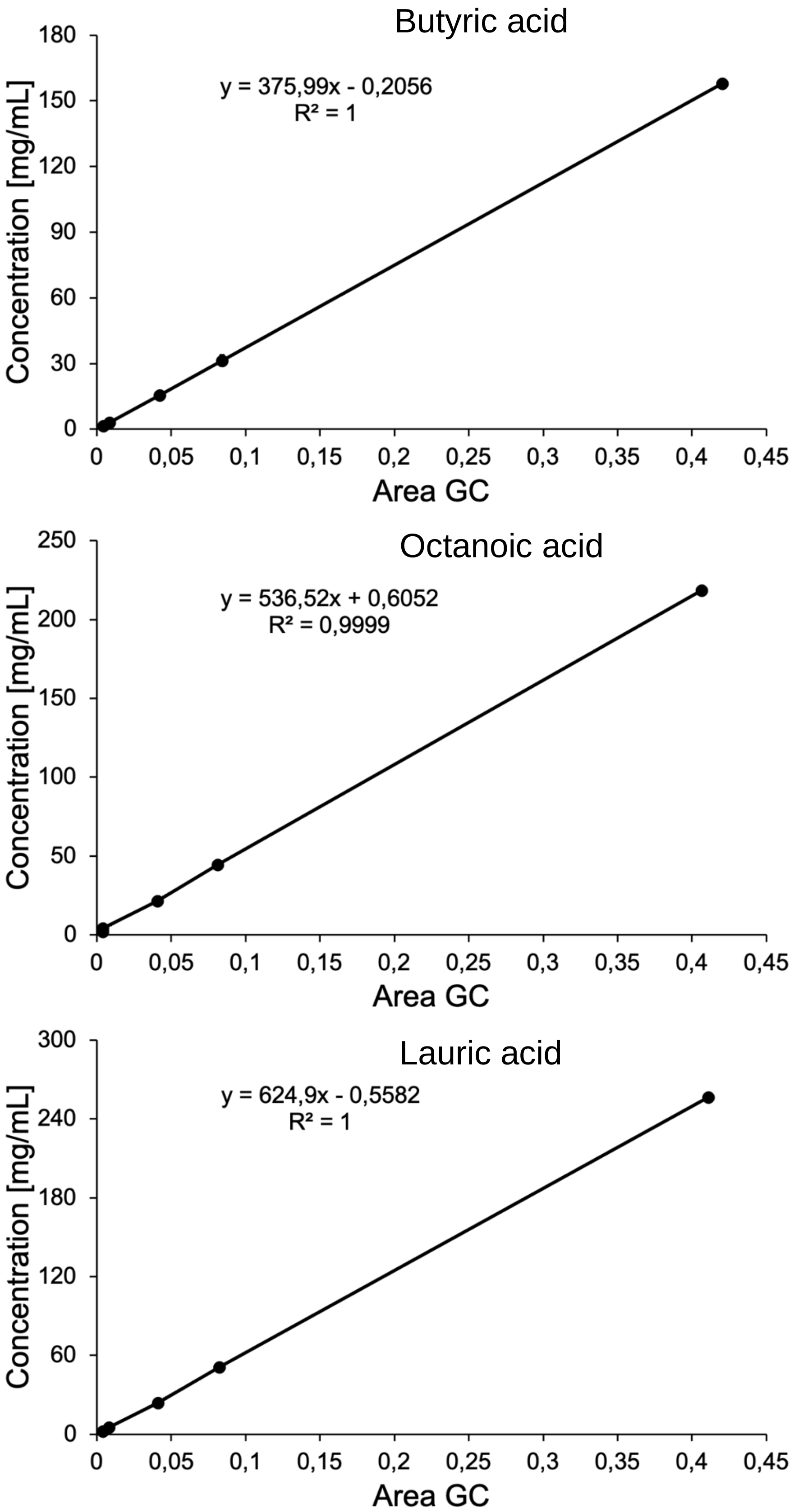

Figure S7. Gas chromatography (GC-FID) calibration curves for butyric acid, octanoic acid and lauric acid.
